# Skewed T-helper (Th)1/2- and Th17/T regulatory-cell balances in patients with renal cell carcinoma

**DOI:** 10.3892/mmr.2014.2778

**Published:** 2014-10-27

**Authors:** LONG LI, CHENG YANG, ZITONG ZHAO, BIN XU, MINGHUAN ZHENG, CHAO ZHANG, ZHIHUI MIN, JIANMING GUO, RUIMING RONG

**Affiliations:** 1 Department of Urology, Zhongshan Hospital, Fudan University, Shanghai 200032, P.R. China; 2 Department of Transfusion, Zhongshan Hospital, Fudan University, Shanghai 200032, P.R. China; 3 Biomedical Research Center, Zhongshan Hospital, Fudan University, Shanghai 200032, P.R. China

**Keywords:** renal cell carcinoma, T helper cells, regulatory T cells

## Abstract

The characterization of CD4^+^ T-cell subsets reflects the immune status and is important in the maintenance of tumorigenesis and homeostasis. To identify changes in the balance of T helper (Th)1, Th2, Th17 and regulatory T cells (Treg) in individuals with renal cell carcinoma (RCC), the present study investigated a total of 131 patients with RCC and 36 healthy volunteers. The number of CD4^+^ T-bet^+^ cells, CD4^+^ GATA binding protein 3^+^ cells, CD4^+^ RAR-related orphan receptor γt^+^ cells, CD4^+^ CD25^hi^ CD127^lo^ CD45RA^−^ cells and CD4^+^ CD25^hi^ CD127^lo^ CD45RA^+^ cells, defined as Th1, Th2, Th17, activated and naïve Treg cells, respectively, were detected in the peripheral blood using flow cytometric analysis. In addition, tumor-infiltrating forkhead box P3 (Foxp3)^+^ cells were examined using immunohistochemistry. Compared with healthy volunteers, a significant decrease in the peripheral percentages of Th1, activated and naïve Treg cells was observed in patients with RCC, while those of the Th2 and Th17 cells were increased. In particular, as the tumor stage and grade progressed, the levels of Th1, activated and naïve Treg cells in the peripheral blood decreased; however, the levels of Th2 and Th17 cells increased. Furthermore, the number of tumor-infiltrating Foxp3^+^ cells increased with increasing tumor stage. These results demonstrated that the balance of Th1 and Th2 cells was skewed towards the Th2 profile and the balance of Th17 and Treg cells was skewed towards the Th17 profile in the peripheral blood of patients with renal cell carcinoma (RCC) and Treg cells were recruited to the tumor sites. Therefore, dysfunctional host anti-tumor immunity was observed in patients with RCC, with a skewed Th1/Th2 and Th17/Treg balance.

## Introduction

Renal cell carcinoma (RCC) accounts for 2–3% of all cancers worldwide and the occurrence rate has increased by 2% per year for the last six decades (1). Metastatic RCC has a particularly poor prognosis, with an overall survival rate of 12 months and a five-year survival rate of <10% (2). Apart from the effects of chemical and physical irritants in the environment, the host immune system is also important in renal carcinogenesis.

Since the cancer immunosurveillance hypothesis (3) was first proposed, the suggestion that the immune system can recognize and eliminate tumor cells has been debated. Several epidemiologic investigations have indicated that immunocompromised patients are more likely to develop cancer, whether it is of viral or non-viral origin, which supports the cancer immunosurveillance hypothesis (4). In certain types of cancer, an increase in the number of regulatory T cells (Treg) advances the progression of cancer by interfering with immune surveillance (5) and exhibiting cancer tolerance (6). However, in specific types of cancer, including colorectal carcinoma, Treg cells have been observed to suppress bacteria-driven inflammation, which promotes carcinogenesis, and thus benefits the host (7).

CD4^+^ T cells are the most important component of the adaptive immune system and have an important effect in host defense against infection and the maintenance of immune homeostasis (6,8). CD4^+^ T cells differentiate into a number of effector subsets, including T helper (Th)1, Th2, Th17 and Treg cells (9). The differentiation of Th1, Th2, Th17 and Treg cells is mediated mainly by T box transcription factor T-bet, GATA binding protein 3 (GATA3), RAR-related orphan receptor (ROR)γt and forkhead box P3 (Foxp3), respectively (9). Th1 and Th17 are involved in the cytotoxic response, while Th2 and Treg mainly suppress the Th1 and Th17-mediated immune response (9). There is a precise dynamic balance in healthy individuals; however, if this balance is lost, the human body becomes vulnerable to infection, autoimmune diseases and tumor growth (10). Enhanced Th2 response accompanied by a decease in Th1 response has been observed in patients with bladder carcinoma (11) and, in oral cancer, the ratio of Th17/Treg cells has been observed to increase in the early stages and decrease in later stages (12).

The Th1/Th2/Th17/Treg-balance in patients with RCC remains to be fully elucidated. Therefore, in the present study, the percentages of Th1, Th2, Th17 and Treg cells were assessed in the peripheral blood of patients with RCC, which was categorized by tumor grade and stage. In addition, infiltrating Treg cells at tumor sites were examined.

## Materials and methods

### Patients and healthy controls

The present study included patients undergoing nephrectomy for non-metastatic RCC between May 2012 and December 2012 at Zhongshan Hospital, Fudan University (Shanghai, China; Table I). The mean age of the patients with RCC and the healthy volunteers was 54.69±13.77 and 48.18±1.78 years, respectively (mean ± standard error of the mean (SEM); P>0.05). Individuals with additional neoplasms, autoimmune diseases, lymphatic or lympho-proliferative disorders were excluded from the study, as were those treated with chemotherapy or immunotherapy prior to surgery. Pre-operative clinical evaluations included a chest X-ray and an abdominal computerized tomography scan. Nephrectomy was performed by an open extraperitoneal/intraperitoneal approach or by laparoscopy, without pre-operative embolization. Peripheral blood (two 5-ml EDTA vials) was obtained from each patient 12 h pre-operatively and from 36 healthy volunteers. Following kidney removal, samples were taken from the neoplasm for immunohistochemical analysis. Approval of the present study was obtained from Zhongshan Hospital’s Ethics Committee (Fudan University, Shanghai, China) and all patients and healthy volunteers provided prior written informed consent.

### Pathological assessment

All tumor specimens were analyzed by two independent pathologists who were experienced in the diagnosis of RCC. The Heidelberg classification (13) was used to assign a histological type to each specimen. RCC was classified according to the 2010 American Joined Committee on Cancer tumor, nodes and metastasis (TNM) staging systems (14) and the Fuhrman nuclear grading system (15).

### Isolation of peripheral blood mononuclear cells (PBMCs)

The peripheral blood, in heparinized tubes, was diluted 1:2 with fresh sterile phosphate-buffered saline (PBS). The PBMCs were then isolated by density gradient centrifugation (611 × g; 25 min) using Histopaque-1077 (Sigma-Aldrich, St. Louis, MO, USA). The viability of the cells was determined using a trypan blue dye exclusion test (Sigma-Aldrich).

### Surface-intracellular staining and flow cytometry

The following anti-human monoclonal antibodies were used: Fluorescein isothiocyanate (FITC)-conjugated mouse anti-CD4 (clone OKT4), FITC-conjugated mouse anti-CD8 (clone HIT8a), allophycocyanin (APC)-conjugated mouse anti-CD25 (clone BC96), APC-conjugated rat anti-RORγt (clone AFKJS-9), phycoerythrin (PE)-conjugated mouse anti-CD45RA (clone HI100), PE-conjugated mouse anti-T-bet (clone eBio4B10), peridinin chlorophyll protein (PerCP)-Cy5.5-conjugated mouse anti-CD127 (clone eBioRDR5) and PerCP-eFluor-conjugated mouse anti-GATA-3 (clone TWAJ). All antibodies were obtained from eBioscience (San Diego, CA, USA). Cells without antibody treatment were used as negative controls. Briefly, the cells were stained with surface markers, fixed and permeabilized with fixation buffer [Dulbecco’s phosphate-buffered saline (pH 7.4) with 4% w/v paraformaldehyde (0.22-μm pore-filtered)]/permeabilization buffer [PBS, 1% fetal calf serum, 0.1% sodium azide, 0.1% saponin (0.2-μm pore-filtered; pH7.4–7.6); eBioscience] and then stained with anti-T-bet, anti-RORγt and anti-GATA-3. Cells (~3×10^5^) were loaded into a BD FACSAria II flow cytometer and data were analyzed using FACSDiva software (v6.1.3; BD Biosciences, Franklin Lakes, NJ, USA). The CD4^+^ T-bet^+^, CD4^+^ GATA-3^+^ and CD4^+^ RORγt^+^ cells were referred to as Th1, Th2 and Th17 cells, respectively. The CD4^+^CD25^hi^ CD127^lo^ CD45RA^−^ cells were referred to as activated Treg and the CD4^+^ CD25^hi^ CD127^lo^ CD45RA^+^ cells as naïve Treg cells.

### Immunohistochemical staining

Immunohistochemical staining was performed on formalin-fixed, paraffin-embedded kidney tissue sections. Human tonsil tissue, provided as formalin/paraformaldehyde-fixed paraffin-embedded tonsil tissue sections by eBioscience, was used as a positive control, while several negative controls were included using renal tissue and omitting certain steps (use of the primary and secondary antibodies) of the staining technique. Using a modified avidin-biotin-peroxidase complex (ABC) method according to Hsu *et al* (16), the samples were stained overnight with monoclonal rat anti-human Foxp3 primary antibodies (1:150; eBioscience) and biotinylated rabbit anti-rat immunoglobulin G secondary antibodies (Abcam, Cambridge, UK). Detection was achieved using ABC-peroxidase solution following peroxidase inhibition and the applied chromogen was diaminobenzidine. The cell nuclei were counterstained using hematoxylin. Non-specific background staining was reduced using DAKO blocking solution (DAKO, Carpinteria, CA, USA) according to the manufacturer’s instructions. The Foxp3^+^ cells were examined (magnification, ×400; TH4-200; Olympus, Tokyo, Japan) in 20 fields of tumor areas and scored semi-quantitatively.

### Statistical analysis

The results obtained from the T-cell subsets are expressed as the mean ± SEM. At the baseline, the proportion gender ratio was compared using a χ^2^ test of independence and the mean age and serum creatinine levels were compared using two-tailed Student’s t-tests. All other variables are presented descriptively. Statistical analysis of the T-cell subsets was performed using a two-tailed independent t-test between two groups and one-way analysis of variance among more than three groups using SPSS 18.0 software (International Business Machines, Armonk, NY, USA). P<0.05 was considered to indicate a statistically significant difference.

## Results

### Proportion of Th1, Th2, Th17 and Treg cells in the CD4^+^ T cells in PBMCs

**Figure 1:**
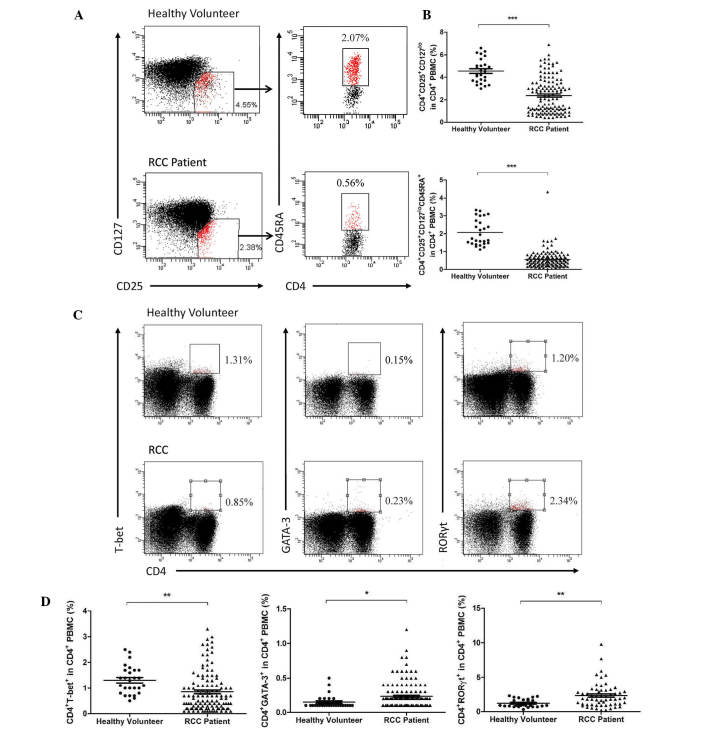
Proportion of Th1/Th2/Th17/Treg cells in CD4^+^ T cells in PBMCs. (A-C) Activated Treg (CD4^+^ CD25^+^ CD127^lo^), naïve Treg (CD4^+^ CD25^+^ CD127^lo^ CD45A^+^) and Th1 (CD4^+^ T-bet^+^) cells were significantly decreased, while (C-D) Th17 (CD4^+^ RORγt^+^) and Th2 (CD4^+^ GATA-3^+^) cells were significantly increased in RCC patients compared with populations in healthy volunteers. Data are expressed as the mean percentage ± standard error of the mean. Th, T helper; Treg, T regulatory; RCC, renal cell carcinoma; PBMC, peripheral blood mononuclear cell; GATA3, GATA binding protein 3; RORγt, RAR-related orphan receptor γt. ^*^P<0.05; ^**^P<0.01; ^***^P<0.001.

To determine the overall changes in CD4^+^ T cells and their subsets in the PBMCs, peripheral blood lymphocytes from patients with RCC and healthy volunteers were stained using the above-mentioned antibodies. A significant decrease was observed in the activated Treg and naïve Treg (P<0.0001) as well as the Th1 (P=0.004) cells (Fig. 1A–C), while the Th17 (P=0.0022) and Th2 (P=0.0317) cells increased compared with those in the healthy volunteers (Fig. 1C and D).

### Proportion of Th1, Th2, Th17 and Treg cells in patients with different stages of RCC

**Figure 2:**
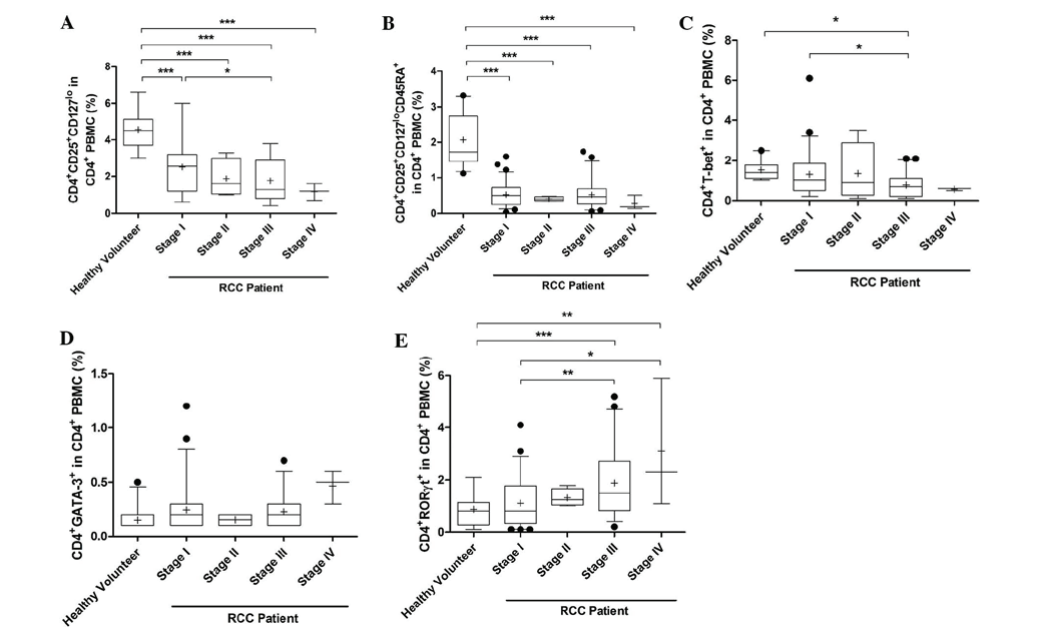
Proportion of Th1/Th2/Th17/Treg cells in different stages of RCC. (A) The percentage of activated Treg (CD4^+^ CD25^+^ CD127^lo^) cells was significantly decreased in stages I-IV (tumor-node-metastasis staging) of RCC patients compared with the healthy volunteers. (B) Percentage of activated Treg cells in stage III was decreased compared with that in stage I. The percentage of naïve Treg (CD4^+^ CD25^+^ CD127^lo^ CD45A^+^) cells in stages I-IV of RCC patients was also decreased. (C) Percentage of Th1 (CD4^+^ T-bet^+^) cells in stage III was decreased significantly compared with in stage I and in the healthy volunteers. (D) Percentage of Th2 (CD4^+^ GATA-3^+^) cells was partially increased in stages I-IV compared with the healthy volunteers, although not significantly. (E) Percentages of Th17 (CD4^+^ RORγt^+^) cells in stages III and IV were markedly increased compared with that in the healthy volunteers. In addition, the proportion of Th17 cells in stages III and IV were higher compared with that in stage I. Data are expressed as the mean ±standard error of the mean. RCC, renal cell carcinoma; PBMC, peripheral blood mononuclear cell; Th, T helper cell; Treg, T regulatory cell; RORγt, RAR-related orphan receptor γt; GATA3, GATA binding protein 3. ^*^P<0.05; ^**^P<0.01; ^***^P<0.001.

The percentage of activated Treg cells was significantly decreased in PMBCs of stage I-IV RCC patients compared with that in healthy volunteers (P<0.0001). In addition, the percentage of activated Treg cells in PMBCs of stage III RCC patients was lower than that in stage I patients (P<0.05; Fig. 2A). The percentage of naïve Treg cells in PMBCs of patients with stage I-IV RCC was also decreased (P<0.0001, Fig. 2B). The percentage of Th1 cells in PMBCs of stage III patients was significantly decreased compared with that in stage I patients and healthy volunteers (P<0.05; Fig. 2C). However, the percentage of Th2 cells was marginally increased in stage I-IV RCC patients compared with that in healthy volunteers, but no significant differences were observed (Fig. 2D). The percentage of Th17 cells in stage III and IV patients was markedly increased compared with that in healthy volunteers (stage III, P<0.0001; stage IV, P=0.0001; Fig. 2E). In addition, the proportion of Th17 cells in stage III and IV patients was higher than that in stage I patients (stage III, P=0.008; stage IV, P=0.002).

### Proportion of Th1, Th2, Th17 and Treg cells in different grades of RCC

**Figure 3:**
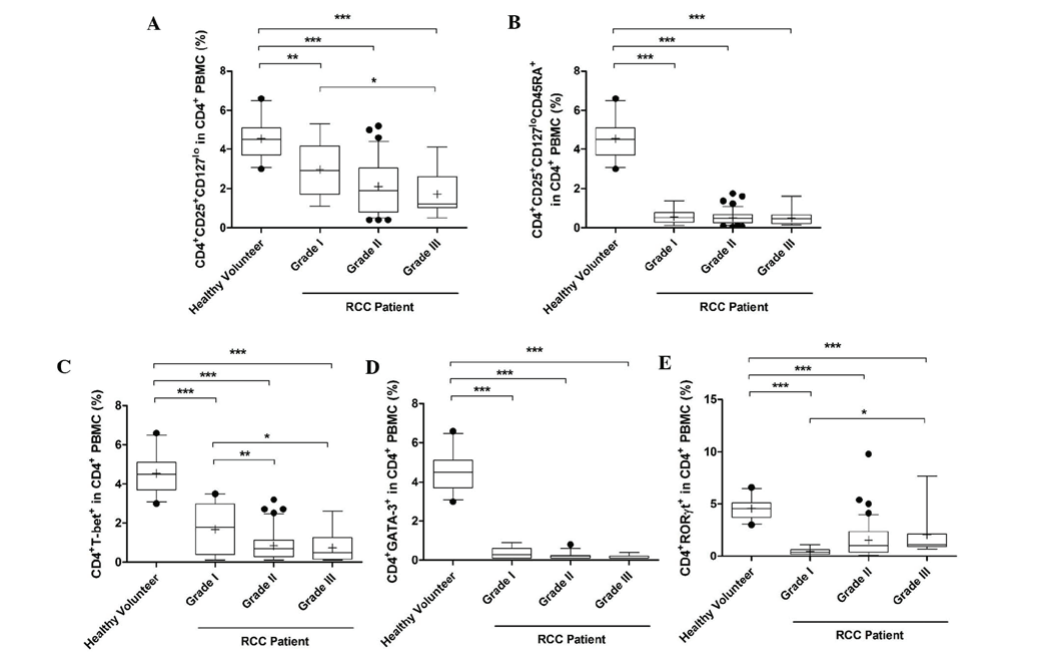
Proportion of Th1/Th2/Th17/Treg cells in different grades of RCC. (A) Proportion of activated Treg (CD4^+^ CD25^+^ CD127^lo^) cells reduced significantly in grades I-III of RCC compared with healthy volunteers. Furthermore, it reduced with increased grade and the proportion in grade III was significantly lower than in grade I. The percentages of (B) naïve Treg (CD4^+^ CD25^+^ CD127^lo^ CD45A^+^) and (C) Th1 (CD4^+^ T-bet^+^) cells in grades I-III were also decreased and (C) the percentage of Th1 cells in grades II and III was significantly lower than that in grade I. (D and E) Percentages of Th2 (CD4^+^ GATA-3^+^) and Th17 (CD4^+^ RORγt^+^) cells in grades I-III were increased significantly compared with the healthy volunteers. (E) Percentage of Th17 cells was also increased in grade III compared with grade I. Data are expressed as the mean ± standard error of the mean. RCC, renal cell carcinoma; PBMC, peripheral blood mononuclear cell; Th, T helper cell; Treg, regulatory T cell; GATA3, GATA binding protein 3; RORγt, RAR-related orphan receptor γt. ^*^P<0.05; ^**^P<0.01; ^***^P<0.001.

The proportion of activated Treg cells was reduced significantly in PMBCs of grade I-III RCC patients compared with that in healthy volunteers (grade II and III, P<0.0001; grade I, P<0.05). Furthermore, this proportion was reduced as the grade increased and the proportion in grade III patients was significantly lower than that in grade I patients (Fig. 3A). Similarly, the percentage of naïve Treg and Th1 cells in grades I-III was also decreased (P<0.0001; Fig. 3B and C). In addition, the percentage of Th1 cells in grades II and III was significantly lower than that in grade I (P<0.01 and P<0.05, respectively; Fig. 3C). However, the percentage of Th2 and Th17 cells in grades I-III was increased significantly compared with that in healthy volunteers (P<0.0001; Fig. 3D and E). The percentage of Th17 cells was also increased in grade III patients compared with that in grade I patients (Fig. 3E).

### Foxp3 immunostaining in tumors at different stages

**Figure 4:**
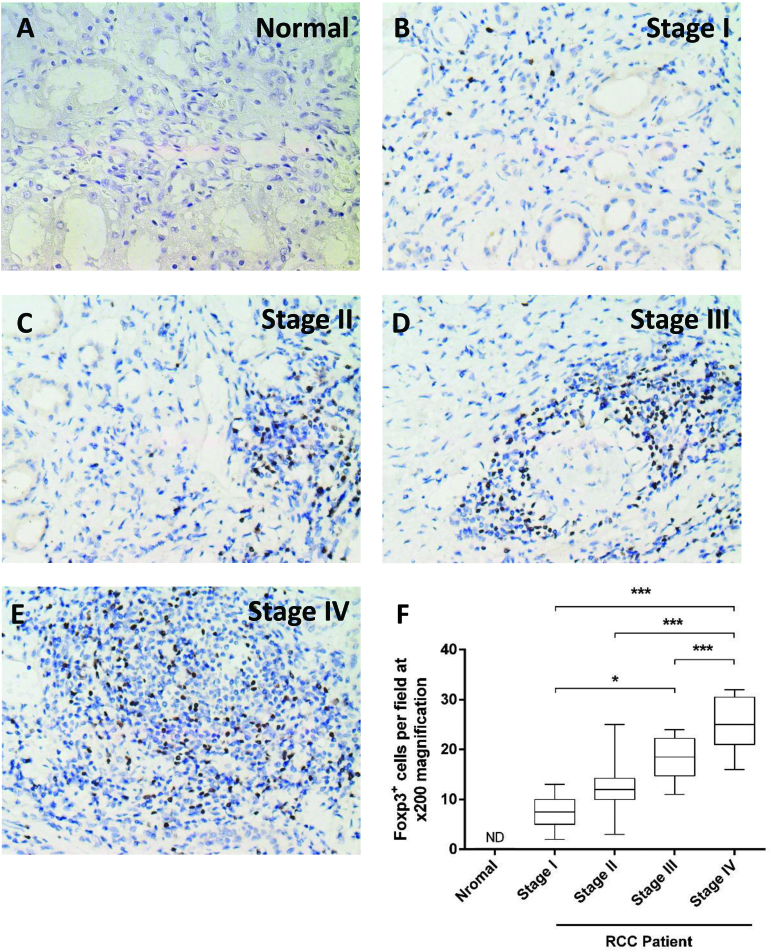
Immunostaining Foxp3 in normal kidneys and different tumor stages. (A) Expression of Foxp3 was not observed in the normal kidneys (A-E) The number of Foxp3^+^ cells in the tumor tissue increased between stages I and IV and the number of Foxp3^+^ cells was significantly higher in stage IV than in stages I-III. In addition, (F) Foxp3^+^ was also increased significantly in stage III compared with stage I. Data are expressed as the mean ± standard error of the mean. RCC, renal cell carcinoma. ^*^P<0.05; ^***^P<0.001.

In order to determine tumor infiltration by Treg, Foxp3 immunostaining was performed (Fig. 4A–E). The number of Foxp3^+^ cells in the tumor samples increased from stage I to stage IV. Furthermore, the number of Foxp3^+^ cells was significantly higher in stage IV compared with that in stages I-III (P<0.0001). The number was also significantly higher in stage III compared with that in stage I (P=0.002; Fig. 4F).

## Discussion

The change in CD4^+^ T cells is representative of the immune status and is important for tumorigenesis and in maintaining homeostasis (12). In the present study, a shift from Th1 toward Th2 cells was observed in patients with RCC, which reflected a skewed balance between the Th1 and Th2 profiles. The cytokines produced by Th1 and Th2 cell subsets are important for the anti-tumor immune function (11). Th1 cells produce type 1 cytokines, including interleukin (IL)-2 and interferon-γ, which exert potent anti-tumor effects and activate cytotoxic lymphocytes (CTL) and natural killer (NK) cell-mediated cytolytic function, associated with effective anti-tumor defense mechanisms (17). Type 2 cytokines, including IL-4 and IL-10, downregulate the tumor-specific immune response by directly suppressing the production of Th1 cytokines, which prevents CTL and NK cell activation and by inhibiting tumor antigen presentation by antigen-presenting cells (18,19). Immune dysfunction in tumor patients includes a skew from a Th1 to a Th2 response, which impairs T-cell immunity towards the tumor (18,20).

In the present study, the skewed proportion of Treg/Th17 cells was associated with the severity of RCC, which was categorized by its stage and grade. This observation was similar to that reported by certain previous studies. In breast cancer, the complete response of breast carcinoma to neoadjuvant chemotherapy was associated with a disappearance of tumor-infiltrating Foxp3^+^ Treg cells (21). However, controversy remains in other studies. Wang *et al* (22) demonstrated an increase in the percentage of Th17 and Treg cells in the circulation of patients with colorectal adenoma and carcinoma. In addition, the percentage of Th17 cells in the circulation increased during early stages; however, Treg cells increased only in advanced stages (23). A possible reason for these differences in results in the circulation may be due to a small sample size (<40 patients in each tumor group). However, rather than the absolute Treg number, its localization pattern has been associated with cancer prognosis (23). Liotta *et al* (24) examined 30 patients with RCC and found that the frequency of Treg cells was significantly higher in tumor-infiltrating lymphocytes than in the peripheral blood, whereas no significant difference was observed in the peripheral blood between healthy volunteers and patients.

The present study revealed a reduction in activated and naïve Treg cells in the peripheral blood, which may be due to the majority of Treg cells being recruited into the tumors. Similarly, the infiltration of Th17 cells which possess an anti-tumor function may be inhibited by tumors; therefore, the proportion of these cells in the PBMCs was increased. It has been demonstrated that tumor cells secrete certain chemokines to recruit Treg cells in order to suppress the attack of cytotoxic T cells in the tumor microenvironment. A study of 31 patients with colon adenocarcinoma revealed that CCR4^+^ CTLA4^hi^ Treg cells accumulated in the tumors. However, there were decreases in the frequencies of activated conventional Th1 cells due to a decrease in the Th1-associated chemokine receptor CXCR3 in the tumors (25). In addition, Chen *et al* (26) demonstrated that the CCL20-CCR6 axis mediated the migration of circulating Treg cells into the tumor microenvironment, which resulted in tumor progression and poor prognosis patients with hepatocellular carcinoma. In the present study, infiltrating Treg cells were also detected using Foxp3 immunostaining. The number of infiltrating Treg cells increased as the tumor stage increased, which suggested that the circulating Tregs were recruited into the tumor and impaired host immunity.

The role of Th17 cells in tumor immunology can be dichotomous. Th17 cells appear to be important in tumorigenesis and in the eradication of an established tumor. A potential protective effect of Th17 has been demonstrated in types of cancer affecting the mucosal tissues, including gut, lung and skin cancer (27,28). In addition, an increase in the number of Th17 cells was observed in the peripheral blood, tumor microenvironment and tumor-draining lymph nodes of several different types of human and mouse tumor (29), including ovarian cancer (30). A previous study on prostate cancer demonstrated that the infiltration of Th17 cells into the tumor was inversely correlated with the Gleason score (31). This implies that Th17 cells mediate an anti-tumor effect in the development of prostate cancer. In the present study, a decrease in the proportion of Treg cells in the peripheral blood was associated with increased RCC grade and a reverse correlation was observed in Th17 cells. These results suggested that increases in Treg cell recruitment with reduced Th17 infiltration may affect the prognosis.

Of note, the plasticity of CD4^+^ T-cell subsets has been demonstrated. These T helper cells, which were previously considered to be the terminal differentiation state, often have the capacity to redirect their functional programs, including the ability of Treg and Th17 cells to convert into each other in certain specific microenvironments (9). Therefore, whether the changes in CD4^+^ T-cell subsets in RCC patients are the cause of tumorigenesis or the consequence of tumor development requires further investigation.

In conclusion, the present study demonstrated that the skewed immunological balance among Th1, Th2, Th17 and Treg cells was distinct in healthy volunteers and an impaired balance was associated with RCC tumor grade and stage. These findings provide novel data to aid in the understanding of the pathological immune status in patients with RCC and assist in the modulation of strategies for potential anti-tumor immunity therapies in these patients.

## Figures and Tables

**Table I:** Patient characteristics.

Characteristic	RCC patients (n)	Healthy volunteers (n)	P-value
Gender			
Male	93	21	>0.05
Female	38	15	>0.05
Fuhrman grade			
I	20	-	-
II	86	-	-
III	25	-	-
IV	0		
TNM stage			
I	65	-	-
II	4	-	-
III	59	-	-
IVw	3	-	-
Pathology			
Clear cell	114	-	-
Cystic papillary	12	-	-
Chromophobe	5	-	-

TNM, tumor-node-metastasis; RCC, renal cell carcinoma.
